# CuboCube: Student creation of a cancer genetics e-textbook using open-access software for social learning

**DOI:** 10.1371/journal.pbio.2001192

**Published:** 2017-03-07

**Authors:** Puya Seid-Karbasi, Xin C. Ye, Allen W. Zhang, Nicole Gladish, Suzanne Y. S. Cheng, Katharina Rothe, Jessica A. Pilsworth, Min A. Kang, Natalie Doolittle, Xiaoyan Jiang, Peter C. Stirling, Wyeth W. Wasserman

**Affiliations:** 1 University of British Columbia, Vancouver, British Columbia, Canada; 2 Department of Medical Genetics, University of British Columbia, Vancouver, British Columbia, Canada; 3 BC Children’s Hospital Research Institute, Provincial Health Services Authority and University of British Columbia, Vancouver, British Columbia, Canada; 4 Centre for Molecular Medicine and Therapeutics, University of British Columbia, Vancouver, British Columbia, Canada; 5 Department of Molecular Oncology, BC Cancer Agency, Provincial Health Services Authority and University of British Columbia, Vancouver, British Columbia, Canada; 6 Graduate Program in Bioinformatics, University of British Columbia, Vancouver, British Columbia, Canada; 7 Terry Fox Laboratory, BC Cancer Agency, Provincial Health Services Authority and University of British Columbia, Vancouver, British Columbia, Canada; 8 Michael Smith’s Genome Science’s Centre, BC Cancer Agency, Provincial Health Services Authority and University of British Columbia, Vancouver, British Columbia, Canada

## Abstract

Student creation of educational materials has the capacity both to enhance learning and to decrease costs. Three successive honors-style classes of undergraduate students in a cancer genetics class worked with a new software system, CuboCube, to create an e-textbook. CuboCube is an open-source learning materials creation system designed to facilitate e-textbook development, with an ultimate goal of improving the social learning experience for students. Equipped with crowdsourcing capabilities, CuboCube provides intuitive tools for nontechnical and technical authors alike to create content together in a structured manner. The process of e-textbook development revealed both strengths and challenges of the approach, which can inform future efforts. Both the CuboCube platform and the Cancer Genetics E-textbook are freely available to the community.

## Introduction

The Open Educational Resources (OER) movement is a global revolution towards accessible, high-quality education worldwide. The William and Flora Hewlett Foundation, which has robustly supported OER development, provides a definition that highlights the release of the materials under “an intellectual property license that permits their free use or re-purposing by others” [1]. Over two decades, the development of OER-related resources has accelerated, in part highlighted by the evolution of Connexions—founded in 1999 and later renamed to OpenStax College—which focuses on expert creation of open teaching materials [2]. Wikipedia, launched in 2001, has been incorporated into classroom teaching, with student participation in content creation [3,4]. Massive open online course (MOOC) platforms, which took off in 2011, have been widely used for online learning [5]. The impact of OER on education is enormous and growing. In 2013, 45,000 students used OpenStax textbooks, and the number of users reached 210,000 in 2014. In 2015, this number had more than doubled, reaching 550,000. By the end of 2016, a total of 1.6 million students had used OpenStax textbooks [6].

One of the motivations behind OER is to reduce cost and expand access. According to the United States Bureau of Labor Statistics, the price of textbooks in the US rose by over 800% from 1978 to 2012, which is higher than the rise associated with medical services (575%), new home prices (325%), and the consumer price index (250%) [7]. Many students and faculty seek affordable alternatives. As of September 2016, 1,352 colleges and universities in the US and 344 colleges and universities outside of the US use at least one of the textbooks published by OpenStax College, reportedly saving students US$155 million in the 2015–2017 academic years [6].

The global effort to create OER textbooks is recognized and has already delivered some success, but the need for more high-quality OER textbooks is urgent and still largely unmet. As of January 2017, 29 professional-grade OER higher education textbooks have been released by OpenStax College, which aims to offer textbooks for the most heavily attended college courses in the US. The current production model resembles the traditional textbook production process—slow, with little turnover—and may not be suitable for education in rapidly evolving scientific fields. Thus, directed participation might be required, possibly through social learning models that derive from the perception that understanding emerges from interactions about content [8]. Many learners benefit from an interactive experience, but most OER textbooks lack support for emerging approaches to social learning. It is our expectation that, when implemented appropriately, the creation of OER e-textbooks should be able to both lower cost and improve learning.

To contribute to the OER movement and to demonstrate the use of an open-access platform towards the creation of OER, we first developed an online textbook creation platform, CuboCube, and then student authors tested and assessed the system by creating an e-textbook. (The mathematical term cubocube refers to a number taken to the third power two times [${\text{n}}^{3}{}^{{}^{3}}$], which we believe reflects a universe of complex educational spaces that is enabled within the CuboCube system. A world of worlds.) While in this report we focus attention on aspects of CuboCube to enable readers to understand the experiences of students in this project, it is not our intention to promote CuboCube over alternatives. CuboCube aims to facilitate textbook development and promote social learning. The system was developed to replace a Wikipedia-based social learning component of an advanced cancer genetics course (Medical Genetics [MEDG] 421) at the University of British Columbia. CuboCube serves two purposes: students can learn and create content for an advanced cancer topic of their interest, and the created content can be reused and updated by future students enrolled in similar courses. The system was beta-tested by 35 students in 2013, and the content was subsequently revised and expanded by a 31-student cohort in 2014 and a 23-student cohort in 2015. (The e-textbook should be cited as follows: Students of UBC Medical Genetics 421, “Cancer Genetics E-textbook”, in P. Seid-Karbasi et al., PLoS Biology, 2017, 15: e2001192. doi: 10.1371/journal.pbio.2001192.) CuboCube provides an intuitive system for content development with a rich text editor and several tools to incorporate content from social media. The end product can be released under multiple licenses based on author/creator preference. Readers can provide comments on the content in each section and suggest additional, relevant social media resources to enhance learning. If a textbook project is created under the Creative Commons Attribution License, readers can directly and simultaneously edit the textbook for improvement. CuboCube adopts a grassroots model that allows any group to develop an e-textbook, and the Cancer Genetics E-textbook illustrates a sustainable model, with respect to both financial concerns and content, for OER creation and update.

## Results

### CuboCube system overview

The CuboCube system was developed as described in S1 Text: Materials and Methods. The website is accessible at http://www.cubocube.com, and the open-source code (released under the permissive Massachusetts Institute of Technology [MIT] license) can be obtained from a GitHub repository (https://github.com/cubocube/website). An end user can be a reader, a participating writer (e.g., a student), or a leader (e.g., an editor or instructor). Each of these categories is granted a role-appropriate capacity to make changes. The base user interface remains constant, with additional features enabled for each user level. The system will be described by a walkthrough of the features.

Currently, a user can have access to existing OER textbooks by clicking the links at the bottom of the CuboCube Home page (http://cubocube.com/): these are the Cancer Genetics E-textbook created for CuboCube and a pre-existing Open Genetics e-textbook that was imported into the system [9]. A registered user can save selected textbooks for quick access by using “My CuboBooks” and view the list of hosted textbooks via “Find CuboBooks” from the Menu in the dashboard. Users can send private messages to other users by using the “new messages” tab next to the “CuboBooks” tab. The “Logged in as” tab allows a user to modify his or her name, email address, and other meta-information.

**Fig 1:**
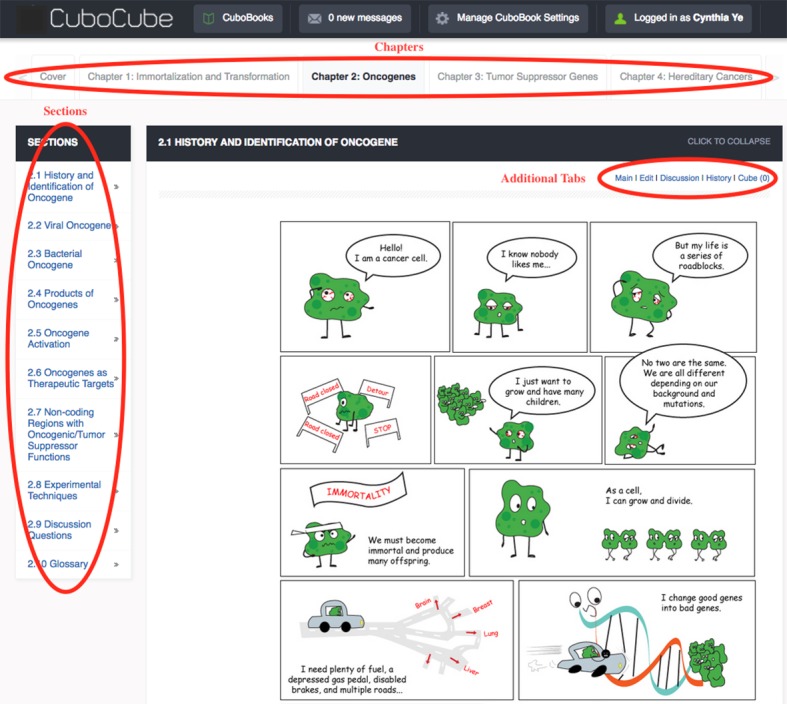
Reader interface of a CuboCube site. Screenshot of a page from the Cancer Genetics E-textbook. A reader can navigate through chapters by using tabs on top of the page and navigate through sections within a chapter using tabs on the left of the page.

All textbooks within the CuboCube framework share the same overarching structure: a textbook contains multiple chapters, each chapter can be divided into sections, and one or several subsections can be added to a section (Fig 1). A list of tabs is designed to facilitate navigation through chapters. Additional tabs are presented within each subsection: Main, Edit, Discussion, History, and Cube. Main shows the current version of the subsection content. A registered reader can access the Discussion board to leave comments and exchange ideas with other users. The History display reports the record of modifications to the subsection. Cube is a function inspired by the “Like” button in Facebook, through which users can inform editors about the perceived quality of the subsection. Both the Discussion and Cube tabs are designed to promote an instant exchange of learning experiences. Nonregistered users cannot edit, participate in discussions, or view the history of changes, but they can access the content.

### Editor interface overview

An editor can utilize the Edit function to contribute to an e-textbook. The rich text editor, CKEditor, is intuitive to use and offers tools to integrate figures and hyperlinks. CuboCube allows multiple individuals to edit the same document simultaneously. If conflicts are detected, one version would present in the Main page, but all versions would be stored and recorded in the History page. The changed areas are highlighted, and editors can discuss to finalize the content. CuboCube’s editor interface is streamlined and intuitive; most students master the system after a 5-minute walkthrough. This feature allows CuboCube to be adopted well by nontechnical users since minimal training is required to use the system.

### Features for leaders

**Fig 2:**
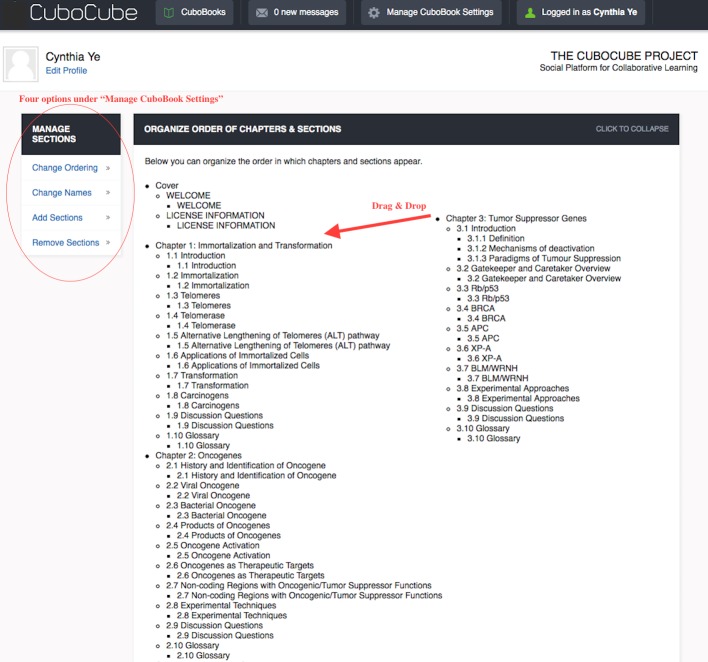
Textbook organization interface for instructors. Authors have the capacity to drag and drop sections to reorganize the content of the e-textbook.

In addition to the aforementioned features, a leader can make structural changes to the textbook, and an additional tab entitled “Manage CuboBook Settings” appears on top of the page. Three options are listed in the dropdown list. On the Manage Sections page, four tabs can be found on the left side bar (Fig 2). By dragging and dropping a chapter or a section to the place of interest, one can rearrange the book quickly. These features make customization of an OER textbook relatively easy and quick. Leaders are provided with the capacity to limit editors to make changes to specific chapters or sections.

### Cancer genetics e-textbook project

To confirm the utility of the editor interface for efficient crowd editing and to assess the crowdsourcing model of textbook development, a 3-year e-textbook project was launched in January 2013. This e-textbook has been released under the Creative Commons Attribution license (CC BY) and is freely accessible to the world.

MEDG 421, Genetics and Cell Biology of Cancer, is a fourth-year honors-style undergraduate course at the University of British Columbia (UBC). Previously, a group project based on the Wikipedia framework was a critical component of the course used to prepare students to contribute to the social knowledge reservoir. CuboCube was implemented to replace the Wikipedia component of the course to offer students a more efficient editing platform and a chance to contribute to an OER textbook that could be used worldwide. During each term, domain experts presented lectures on key cancer genetics topics to the class. Each student was assigned to 2–3 chapters focused on a key concept, with 4–5 students per chapter. The teams worked collaboratively to create and/or update content based on information from the lectures and additional information from scientific literature. Students could use either a “Plan/Discussion” section, which was deleted upon completion, or the Discussion board to coordinate within the group and between groups to reduce redundancy and to improve the flow of the textbook. The process is self-directed and requires students to collaborate extensively.

A key challenge in the creation of learning materials is the development of graphics. An artist (at the time a student in a local illustration training program) was hired to develop images for the project. Initially, student-created images were expected to be sufficient, but confirming the originality of the images and a desire to provide graphical continuity across the book motivated the recruitment of the artist for a subset of images. Two templates to illustrate timeline and balance were produced and are available for future adaptation. The illustrator created 30 conceptually important images and released those under the Creative Commons Attribution-ShareAlike 4.0 International license (CC BY-SA 4.0).

The end product is a cancer genetics textbook with ten chapters developed by 89 students: Chapter 1: Immortalization and Transformation; Chapter 2: Oncogenes; Chapter 3: Tumor Suppressor Genes; Chapter 4: Hereditary Cancers; Chapter 5: Cancer Stem Cells; Chapter 6: Personalized Cancer Therapy; Chapter 7: Angiogenesis; Chapter 8: Apoptosis and Senescence; Chapter 9: Metastasis; and Chapter 10: Cancer Genome. The textbook covers basic cancer genetic principles but also shows the current trend of cancer research. During the development period (2013–2016), the CuboCube system received 62,781 unique visits.

## Discussion

### Principal findings

Three successive engaged cohorts of undergraduate students cooperatively acted to develop and refine the Cancer Genetics E-textbook. They utilized a novel software system, CuboCube, which provides an intuitive and flexible OER textbook development system to enable textbook creation and customization and supports social learning. It is intended to allow nontechnical users to develop textbooks online with minimal training and technical skill. The student efforts were supplemented by a set of images provided by an artist, which provide graphical continuity across the project. The effort affirms the capacity of a structured group-sourced project to create free textbooks and suggests that coordinated global efforts could accelerate the development of quality OER e-textbooks.

There are two major models for OER textbook construction: a public model represented by the Wikibooks project and a private model represented by the OpenStax College project [10]. These two models represent the two ends of a continuum. The large number of users in Wikibooks allows for fast content development: 2,867 books with 55,239 pages had been created in English by September 2015 [11]. However, the lack of peer review and the unknown expertise of content creators have been noted as concerns [12]. The debate around the accuracy of open content editing is ongoing, but Wikipedia has been shown to have similar content quality compared to material created in a formal peer-reviewed model [13]. The content quality may be improved when participating authors realize that an international community recognizes the value of their contributions [8]. OpenStax College has created 29 professional-level OER textbooks (as of January 2017), which have been adopted by many universities and colleges. A small group of staff, including a technical support team and editors, works closely with the author(s) following a more traditional textbook construction model. The learning curve to master the Connexions system and the lack of basic collaborative features may contribute to scalability issues with such closed models [14].

The CuboCube system allows for adoption based on author preference of models that lie anywhere within the broad spectrum. As demonstrated by the Cancer Genetics E-textbook project, simultaneous editing from multiple users allows for textbook construction harnessing the power of the crowd. CuboCube also allows for remixing and reuse of existing content in the system under the Creative Commons license and is highly customizable to meet the needs of instructors. This approach is suitable for creating textbooks on domain subjects that are studied by a small group of students in a single institution but interest a substantial global population. Also, a subject that is under rapid evolution can benefit from using a textbook that can be updated quickly.

We recognize the challenges faced by OER in motivating creators and making any effort sustainable [10,12]. An investigation of over 50 high-quality Wikibooks shows that over 50% of the content for most books is written by a small core of authors [15]. Authors can choose a suitable type of license within the CuboCube system, and this flexibility has the potential to encourage more authors to join into the process of e-textbook development. Such community participation circumvents expenses imposed by hiring a professional editing team, but it remains unclear when professionals become needed. Within this project, an artist provided a more consistent illustration set for the project. Supporting such targeted compensated contributions can improve quality if even modest funds are available. The aforementioned features of CuboCube make it a better model to satisfy the need of a class, especially the easiness of remixing and rearranging existing material over time and of controlling the editorial access to subsets of the material.

The potential benefits to students of low-cost student-created learning materials are many. Several advantages have already been established. Students who use OER can achieve the same learning outcomes and save a significant amount of money [16]. In some cases, the learning material cost savings may allow students to enroll for a higher number of credits, resulting in faster completion of degrees [17]. In developing the Cancer Genetics E-textbook, we recognize that the creation process has an additional benefit to learning. To master a field of knowledge, a student needs to go beyond “learning about” the subject matter and start “learning to be” a full participant in the field [8]. Participating in textbook development allows students to engage in “learning to be” even as they are trying to master the content of a field. This process encourages students to practice their skills to seek knowledge when needed for a particular task and engage in self-directed learning [8]. Although chapter titles were set by instructors for the Cancer Genetics E-textbook, students were free to choose the specific topics they wanted to work on and decide the appropriate depth and breadth after their own independent study. Moreover, students worked in groups to construct the cancer e-textbook, and peer interactions may have promoted a greater interest in and responsibility for their own learning [8,18]. Light found that students’ ability to form or participate in small study groups was one of the strongest determinants of students’ success in higher education [8,19]. The utilization of such a system to create a textbook enhances collaboration and the social learning experience for students by providing easy access to a discussion board associated with the material and the “Cube” function to reinforce a sense of participation.

Student-led course material development is a well-established approach in education [20]. For instance, a traditionally formatted textbook in forensic anthropology was created by students at the University of Dundee [21]. Recently, a biochemical methods e-textbook was developed by undergraduate students at the University of Oklahoma, using iBooks Author (Apple) and subsequently transferred to a Web-based version for broader access [20]. As CuboCube allows simultaneous modification by users, it should broaden the capacity for class-driven textbook creation. Moreover, student-led course material development allows up-to-date modification of the context, making the OER sustainable over time.

The issue of copyright and intellectual property will be an ongoing challenge for community-created resources. The rise of the Internet has made plagiarism and copyright issues more widespread than before [22]. Completed chapters in the Cancer Genetics E-textbook were analyzed with the TurnItIn Originality Check system. With figures, originality confirmation is more complicated. In this project, we noted a tendency for students to use existing figures with a citation but not with confirmed creator approval. We removed such figures in a final review of the material. The quality of student-made figures is limited by students’ illustration skills. Considering the value of accurate and interesting scientific figures for the scientific communities, as noted above we hired a student illustrator to create a set of figures for key concepts. In the long-term, open-use image and figure archives such as Wikimedia Commons will be critical to advancing projects like CuboCube. To be consistent with the license terms for Wikimedia Commons, we released the figures for the Cancer Genetics E-textbook under the CC BY-SA license. We hope to find more permissive ways to share figures in the future, as OER benefits from the least restrictive licenses being utilized.

### Future directions

We are seeking collaborators who teach cancer genetics to enrich and improve the Cancer Genetics E-textbook, ideally by bringing together cohorts of students around the world to develop a second edition. Such collaborative efforts allow us to provide an opportunity for teacher collaboration on curriculum development [23]. A flexible open-source system is ideal to facilitate institutional collaboration, either for formal or informal learning. Dissemination of such a system to a wider audience and exploration of its utility within other settings will help identify and develop key features desired by users to meet teaching and learning needs, which will contribute to the OER movement.

## Supporting information

S1 Fig Entity relationship diagram for CuboCube system provides an overview of the database structure. (TIFF)

S1 Text Materials and Methods. (DOCX)

## Abbreviations

CC BY: Creative Commons Attribution license
CC BY-SA 4.0: Creative Commons Attribution-ShareAlike 4.0 International license
MEDG: Medical Genetics
MIT: Massachusetts Institute of Technology
MOOC: massive open online course
OER: Open Educational Resources
UBC: the University of British Columbia
