# Supplementary figures and images for: CuboCube: Student creation of a cancer genetics e-textbook using open-access software for social learning
Source: PLoS Biol. 2017 Mar 7;15(3):e2001192. doi: 10.1371/journal.pbio.2001192 (PMC5340349; doi:10.1371/journal.pbio.2001192)

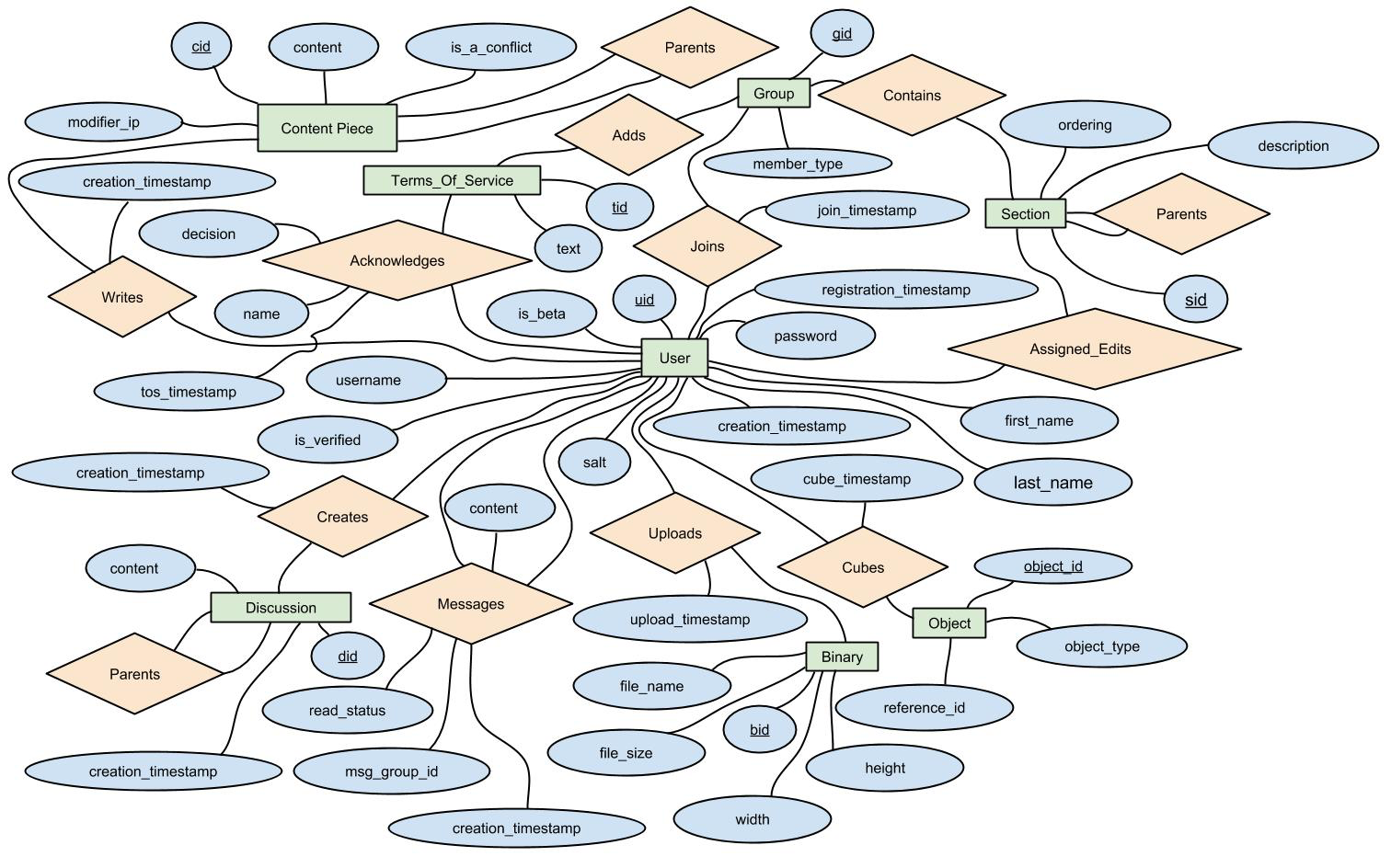

Supplement: S1 Fig — (TIFF) [file pbio.2001192.s001.tiff]
