# Supplementary material for: CuboCube: Student creation of a cancer genetics e-textbook using open-access software for social learning
Source: PLoS Biol. 2017 Mar 7;15(3):e2001192. doi: 10.1371/journal.pbio.2001192 (PMC5340349; doi:10.1371/journal.pbio.2001192)
Supplement: S1 Text — (DOCX) [file pbio.2001192.s002.docx]

**Supplement**

**Materials and Methods**

***System Design***

CuboCube’s architecture consists of four primary layers: Linux, Apache, MySQL and PHP- popularly known as the LAMP stack. CuboCube resides on a single Amazon Web Services Elastic Compute Cloud instance. Data persistence is handled by the MySQL layer for text based data storage and retrieval. We store some binary data on the server with the pointers stored in our database. An entity relationship diagram portraying the relationships and entities for CuboCube is shown in Fig S1. Using a flexible, dynamic programming language such as PHP has served well in keeping operational costs low, and allowing us to implement and deploy new features quickly.

We made extensive use of jQuery and other open source Javascript libraries to provide a rich user experience similar to that of a desktop application. This method contrasts with the user experience of more traditional websites where the user has to follow hyperlinks to display new views, and consequently forcing the loading of an entire new page with each click. Our approach has been to minimize the amount of page loads for any user task, loading necessary website data asynchronously (AJAX) and then using that data to render the appropriate view for the user. This approach has tradeoffs. While it enables a more enjoyable user experience, on the downside is an increase in development costs as the addition or modification of features may require changes on both the server-side and client-side code base.

***Content Contributor Workflow***

CuboCube allows students and instructors to collectively write an e-textbook, or CuboBook as it is called within the platform. There are four tiers of user roles for each e-textbook: reader, writer, leader and admin. By default, writer accounts can only view content pieces—chapters and sections. To edit content pieces the writer’s account needs to have been assigned to that specific section or chapter by a leader or admin account. In the past, per instructor (leader) feedback, we have opened up content pieces to be editable by all students (writers) bypassing the section assignment permission system.

***CuboBook Initiation***

We currently do not expose functionality to create a new CuboBook automatically. When interest is expressed for a new e-textbook, we create a new CuboBook within the system and assign the appropriate user as an admin.

***Content Editing***

CuboCube is integrated with a WYSIWYG (“What you see is what you get”) editor called CKEditor. This provides content contributors with a familiar and easy-to-use interface for creating content, without having to worry about the technical aspects involved. Internally, content pieces are stored as HTML markup. Prior to inserting a new content piece into our database we pass the content to our Content Purifier which alters or removes potentially malicious markup. Edited content is saved as a new item, maintaining the previous versions in the system. The system always displays the latest version of the content piece to the end user. Users with edit access can view the history of a section, and see how the content has changed over time. Based on user feedback, we implemented a Diff tool that highlights any text that was either added or removed in a specific revision. We recognize that over time there will be a build up of numerous revisions for any given section. Content contributors are able to mark certain revisions as ‘Important’ and enter an optional explanatory note. They are then able to filter revisions to show only those that are ‘Important’. Lastly, concurrent editing presents its own challenges. Our current strategy is to detect when two or more content pieces originate from the same parent. When they do, a warning is shown to the editor when he or she tries to save a revision indicating a possible conflict. The editor then has to look at the section history and use the Diff tool to manually merge concurrent changes.

***Social Functions***

Users on CuboCube are able to send messages to each other. CuboCube’s messaging system includes a read-status feature, which informs message senders whether the recipient has read their message. Users are able to form discussions around sections within a CuboBook to help enrich the content by either asking questions or leaving a comment pertinent to the content. Content contributors and consumers are able to support positive engagement by “Cubing” comments in which they feel adds positive value. Comments that have the most recent engagement are sorted to be near the top. A specific section revision can also be “Cubed” by users.
